# Performance of an ELISA and Indirect Immunofluorescence Assay in Serological Diagnosis of Zoonotic Cutaneous Leishmaniasis in Iran

**DOI:** 10.1155/2014/505134

**Published:** 2014-08-11

**Authors:** Bahador Sarkari, Marzieh Ashrafmansouri, GholamReza Hatam, Parvaneh Habibi, Samaneh Abdolahi Khabisi

**Affiliations:** ^1^Basic Sciences in Infectious Diseases Research Center, Shiraz University of Medical Sciences, Shiraz, Iran; ^2^Department of Parasitology and Mycology, School of Medicine, Shiraz University of Medical Sciences, Shiraz, Iran

## Abstract

Serological assays have been extensively evaluated for diagnosis of visceral leishmaniasis (VL) and considered as a routine method for diagnosis of VL while these methods are not properly evaluated for diagnosis of cutaneous leishmaniasis (CL). This study aimed to assess the performance of indirect immunofluorescent-antibody test (IFA) and enzyme-linked immunosorbent assay (ELISA) for serodiagnosis of cutaneous leishmaniasis in Iran. Sixty-one sera samples from parasitologically confirmed CL patients and 50 sera from healthy controls along with 50 sera from non-CL patients were collected. Antigen was prepared from promastigotes and amastigotes of *Leishmania major*. IFA was used to detect anti-*Leishmania* IgG while ELISA was used to detect anti-*Leishmania* IgM, total IgG, or IgG subclasses (IgG1 and 4). ELISA, for detection of total IgG and IgM, showed sensitivity of 83.6% and 84.7% and specificity of 62.7% and 54.6%, respectively. Sensitivity and specificity of ELISA for detecting IgG1 and IgG4 were 64%, 75% and 85%, 49%, respectively. Sensitivity and specificity of IFA were 91.6% and 81%. *Conclusion*. Findings of this study demonstrated that serological test, especially IFA, can be used for proper diagnosis of CL.

## 1. Introduction

Leishmaniases are a serious health problem across the world. It is estimated that there are about 12 million infected people throughout the world [1].

Both cutaneous and visceral leishmaniasis are present in Iran and cutaneous leishmaniasis (CL) has a vast distribution, being found in almost all provinces, especially in Fars, Isfahan, and Kerman [2–6].

The laboratorial diagnosis of CL is mainly based on parasitological method which searches for the parasite in the lesions or culture of the lesion material. Although these methods are specific, they suffer from the low sensitivity, the possibility of culture contamination, and also the difficulties of parasite growth. Along with parasitological method, molecular techniques have been used for the diagnosis of CL. However, the technical requirements, the relatively high cost, and also the persistence of parasite in the lesion after the treatment hindered its routine applicability [7–10].

Serological assays have been extensively evaluated for diagnosis of visceral leishmaniasis (VL) and considered as a routine method for diagnosis of VL while these methods are rarely used for diagnosis of CL. In recent years, serological approaches have been evaluated for diagnosis of CL in few studies [11–17].

Among the serological tests, indirect immunofluorescence (IFA), Direct Agglutination Test (DAT), and enzyme immunoassay (EIA) are the most frequently evaluated tests for diagnosis of mainly American cutaneous leishmaniasis [12, 14, 16].

However, such approaches have not been properly evaluated for diagnosis of zoonotic cutaneous leishmaniasis (ZCL). Lack of such information, especially in Iran, justified the conductance of the current study which aimed to determine the efficacy of two serological assays, ELISA and IFA, for diagnosis of cutaneous leishmaniasis in Iran.

## 2. Materials and Methods

### 2.1. Serum Samples

Sera samples were collected from sixty-one parasitologically confirmed CL patients referred to Shiraz hospitals. For parasitological diagnosis, a sample was taken from the skin lesion of each suspected case. With each case, the border of the skin lesion was slit with surgical lancet and a tissue scraping from the slit was smeared on a clean glass slide, fixed with methanol, and then stained with Giemsa's stain. Each such smear was carefully examined by light microscopy under oil immersion and the diagnosis of CL was confirmed by the positive smear for Leishman bodies. Furthermore, samples prepared from the lesion of the patients were analyzed by PCR to find out the species of the parasite. Control samples (*n* = 50) were obtained from healthy individuals with no history of CL. Moreover, 50 sera samples were collected from non-CL patients. Collected samples were tested by IFA and ELISA. Ethical approval of the study was given by the Ethics Committee of Shiraz University of Medical Sciences, and consent was obtained from the participants.

### 2.2. Preparation of Antigens

Amastigotes of* L. major* (MRHO/IR/75/ER) were cultivated as described before [18]. The parasites were washed three times (1500 ×g for 20 min) with PBS. Protease inhibitors were added and the parasites were resuspended in PBS and submitted to freeze-thawing followed by sonication. The lysed material was centrifuged (1500 ×g for 20 min at 4°C) and the supernatant was removed; the protein content was estimated and the extracted antigen was stored at −20°C until the use in ELISA. Moreover, the promastigotes of* L. major* (same strain as above) were propagated in RPMI media. Antigen slides for IFA were prepared by washing the promastigote cells thoroughly with PBS and by loading 20 *μ*L of cell suspension at a density of 5 ∗ 10^5^ cell/mL onto each spot of a multiple spot Teflon slide. The slides were allowed to dry at room temperature and stored at −20°C until use.

### 2.3. Enzyme Linked Immunosorbent Assay (ELISA)

To detect anti-*Leishmania* antibodies in sera of CL patients, collected sera were evaluated in an ELISA system. ELISA was carried out in flat-bottom 96-well microplates (Nunc, Nalgene, Nunc International, Roskilde, Denmark). The plates were sensitized with 5 *μ*g/mL of amastigotes antigens (100 *μ*L/well) in coating buffer (0.05 M carbonate-bicarbonate buffer, pH 9.6) and incubated at 4°C overnight.

Excess antigen was removed by washing the plate five times in phosphate buffered saline-Tween 20 (PBST, pH 7.4 containing 0.05% Tween 20). Blocking was made with 3% skimmed milk in PBST for 2 hours. The wells were washed as before and 100 *μ*L of serum samples (1/100 dilution in PBST) from CL patients along with samples from healthy subjects, as negative controls, and sera from non-CL patients were applied to the plates and incubated for 1.5 hour at room temperature. The plates were washed as before and 100 *μ*L of horseradish peroxidase (HRPO)-conjugated antibody against either human IgG or IgM (Abcam) or HRPO-conjugated mouse anti-human IgG1 or IgG4 (GIBCO) in PBST was added to the plates and incubated for 1 hour at room temperature. After washing as before, the plates were incubated with chromogen/substrate (100 *μ*L/well of OPD, 0.025% H_2_O_2_ in 0.1 M citrate buffer, pH 5). The absorbance at 490 nm was checked with an ELISA microplate reader. For each antigen, the cut-off value, which differentiates positive from negative results, was set by defining the cutoff as the mean value of the normal serum group plus three standard deviations.

### 2.4. Indirect Immunofluorescence Assay (IFA)

Indirect immunofluorescence assay was performed by using promastigotes of* L. major*. The serum samples were diluted 1 : 16 in PBS-skimmed milk 2% for preliminary screening and the positive samples were serially diluted up to 1 : 1024. Ten microliters of each diluted serum was placed in the well of the slides and incubated in a humid chamber at 37°C for 30 minutes. Slides were washed in PBS (three times, each 10 minutes), dried, and incubated for 30 minutes at 37°C with fluorescent-conjugated rabbit anti-human IgG (Sigma), diluted 1 : 1000, and Evans blue solution, diluted 1 : 10000. Slides were washed and air-dried. Finally, the samples were observed under immunofluorescent microscope and the titers of 1 : 16 and above were considered as positive. To determine the cutoff that best discriminates the sera of CL patients from the others, sera from CL patients, non-CL patients, and healthy individuals were analyzed and the cut-off point chosen was 1 : 16 titer.

## 3. Results

CL patients consisted of 41 males and 20 females. Mean age of the patients was 37 (aged between 9 and 83 years). Most of the patients (28.3%) were in 20–30-year-age group. Most of patients (37%) had CL lesion in their hands whereas the rest had lesion in their feet (21.3%), faces (13.1%), or trunks (6.4%). Mean number of lesions of the patients was three. Causative agent of CL was* L. major* as determined by PCR.

Using ELISA for detecting of total-IgG against* L. major*, 51 cases of CL patients (83.6%) had a positive reaction in ELISA while 10 cases (16.4%) of healthy controls and 19 cases of non-CL patients were also positive by ELISA. Accordingly, sensitivity and specificity of ELISA for diagnosis of CL were 83.6% (95% CI = 71.4%–91.4%) and 62.7% (95% CI = 52.9%–71.6%), respectively. When the system was used for detecting anti-*Leishmania* IgM, a sensitivity of 84.7% (95% CI = 72.5%–92.3%) and specificity of 54.3% (95% CI = 44.2%–64.6%) were obtained for the assay. The ELISA system was also used for detection of anti-*Leishmania* IgG subclasses (IgG1 and IgG4) and a sensitivity of 64% and 85% was found for IgG1 and IgG4, respectively.

Using IFA, a sensitivity of 91.6% (95% CI = 80.8%–96.8%) and a specificity of 81% (95% CI = 71.6%–87.8%) was found for IFA when the cutoff was set at 1 : 16. However, when the cut-off point was set as 1 : 128, fourteen cases (27.5%) of CL patients remained positive while none of healthy controls or non-CL patients had a positive reaction. Accordingly, the assay showed a low sensitivity but a specificity of 100% when the cutoff was set at 1 : 128 titer.  Table 1 shows the details of the performance of ELISA and IFA in diagnosis of CL in this study.

Statistical analysis of the data showed a fair agreement (kappa = 0.4) between ELISA (for detecting total IgG) and IFA.

## 4. Discussion and Conclusion

Parasitological diagnosis, which relies on detecting of* Leishmania* parasite in lesion or on cultivation of tissue samples of CL patients, still remains as a gold standard for diagnosis of CL [8, 9, 19]. Although the direct microscopic identification of the parasite is simple, its low sensitivity of less than 60% is problematic, particularly in chronic cases where parasitemia is low. Cultivation of tissue samples is a useful parasitological method since it allows the isolation of the parasite identification of the species, but it is time-consuming and culture may get infected by fungi or bacteria.

Different serologic assays such as ELISA, IFAT, Direct Agglutination Test (DAT), and immunoblotting have been evaluated and used for routine diagnosis of visceral leishmaniasis [20–22]. The use of these serologic tools in diagnosis of CL has also been evaluated in a few of studies [23–25]. Nevertheless, no serologic test is yet available for routine diagnosis of CL.

The principal objective of the current study was to evaluate the performance of two common serological methods, ELISA and IFA, in diagnosis of ZCL in Iran. Findings of the study demonstrated an appropriate performance (sensitivity of 91.6%) for IFA in diagnosis of CL. Previous studies which evaluated the efficacy of serological methods in diagnosis of CL have generated variable results. The reported sensitivity for CL is ranging from 23% to 95% [11–16].

Zeyrek et al. reported a sensitivity of 78.4% and specificity of 69.3% for an ELISA system for diagnosis of CL in Turkey [15]. These findings are consistent with the findings of our study. Romero et al. reported a sensitivity of 89% for an ELISA system with antigen of* Leishmania mexicana* and sensitivity of 71% with antigen of* Leishmania braziliensis *[25]. Jensen et al. reported an ELISA test with a sensitivity of 67% in 33* L. major* infected CL patients, by using a sequence specific peptide antigen [24].

In our study a satisfactory performance was found for IFA for diagnosis of CL. Lower sensitivity has been reported by Mosleh (81%) and also by Monroy-Ostria (85.4%) for IFA [23, 26]. In these studies, the cut-off point has been set on 1 : 16 titer and we used a same cutoff for IFA. In our study, when the cutoff raised to 1 : 124, none of control samples remained positive by IFA while more than 20 percent of CL patients were strongly positive by this and also by higher titers (up to 1 : 1028). Considering this cutoff, the specificity of the test is 100% while the sensitivity lowered to 23%.

In the current study, attempt was made to detect anti-*Leishmania* IgG subclasses by ELISA in CL patients. However, the results were not any better than that of the detection of total IgG.

In CL patients, antibody levels are largely considered to be very low. This is not the case in VL where antibodies may reach exceedingly high levels leading to hypergammaglobulinemia which is a feature of the diseases. In addition, seropositivity may also depend on the duration of the disease and also the number of skin lesions [23]. Thus in CL, lower sensitivity of serologic tests can be expected due to low antibody titers. In our study in CL patients, correlation between the positive serological test and duration of the disease was significant (*P* < 0.05) while no association was found between the number of lesions and seropositivity.

Taken together, results of this study demonstrated that serological tests, especially IFA, have an appropriate performance for diagnosis of CL and these tests, along with the parasitological methods, can be used for proper diagnosis of CL.

## Figures and Tables

**Table 1:** Performance of different ELISA and IFA in diagnosis of cutaneous leishmaniasis.

Type of test	Sensitivity (%)	Specificity (%)	PPV (%)^a^	NPV (%)^b^
Total-IgG ELISA	83.6	62.7	55.4	87.3
IgG1-ELISA	64	75	56.1	80.6
IgG4-ELISA	85	49	50	84.4
IgM-ELISA	84.7	54.3	53.2	85.4
IFA	91.6	81	74.3	94.1

^a^Positive predictive value.

^b^Negative predictive value.
